# Antioxidant Activity of the Lignins Derived from Fluidized-Bed Fast Pyrolysis

**DOI:** 10.3390/molecules22030372

**Published:** 2017-03-01

**Authors:** Sohail S. Qazi, Dongbing Li, Cedric Briens, Franco Berruti, Mamdouh M. Abou-Zaid

**Affiliations:** 1 Department of Chemical and Biochemical Engineering, University of Western Ontario, London, ON N6A 5B9, Canada; soq823@mail.usask.ca; 2 Canadian Wood Fibre Centre, Natural Resources Canada, Great Lake Forestry Centre, Sault Ste. Marie, ON P6A 2E5, Canada; 3 Institute for Chemicals and Fuels from Alternative Resources (ICFAR), University of Western Ontario, 22312, Wonderland Road N, Ilderton, ON N0M 2A0, Canada; dli86@uwo.ca (D.L.); cbriens@uwo.ca (C.B.); fberruti@uwo.ca (F.B.)

**Keywords:** antioxidants, value-added chemicals, HPLC-MS/MS, metabolomics, pyrolytic lignins, biomass, bio-oils, phenolics, free-radical scavenging activity, bioactive compounds

## Abstract

A challenge in recent years has been the rational use of forest and agriculture residues for the production of bio-fuel, biochemical, and other bioproducts. In this study, potentially useful compounds from pyrolytic lignins were identified by HPLC-MS/MS and untargeted metabolomics. The metabolites identified were 2-(4-allyl-2-methoxyphenoxy)-1-(4-hydroxy-3-methoxyphenyl)-1-propanol, benzyl benzoate, fisetinidol, phenyllactic acid, 2-phenylpropionic acid, 6,3′-dimethoxyflavone, and vanillin. The 2,2-diphenyl-1-picrylhydrazyl radical scavenging activity (DPPH), trolox equivalent antioxidant capacity (TEAC), and total phenolics content (TPC) per gram of pyrolytic lignin ranged from 14 to 503 mg ascorbic acid equivalents, 35 to 277 mg trolox equivalents, and 0.42 to 50 mg gallic acid equivalents, respectively. A very significant correlation was observed between the DPPH and TPC (*r* = 0.8663, *p* ≤ 0.0001), TEAC and TPC (*r* = 0.8044, *p* ≤ 0.0001), and DPPH and TEAC (*r* = 0.8851, *p* ≤ 0.0001). The polyphenolic compounds in the pyrolytic lignins which are responsible for radical scavenging activity and antioxidant properties can be readily profiled with HPLC-MS/MS combined with untargeted metabolomics. The results also suggest that DPPH, TEAC, and TPC assays are suitable methods for the measurement of antioxidant activity in a variety of pyrolytic lignins. These data show that the pyrolytic lignins can be considered as promising sources of natural antioxidants and value-added chemicals.

## 1. Introduction

Renewable plant biomass holds an eco-friendly and promising future in the years ahead, since it is an effective alternative source of renewable energy in the current scenario of depleting oil reserves, global warming, and growing environmental concerns. The biomass can be converted to other valuable forms of energy by a number of processes such as biological, mechanical and chemical. However, thermal processes (e.g., fast pyrolysis) can yield complex chemicals at a very high yield (75 wt %) [1].

Pyrolysis has been applied for thousands of years for charcoal production and historical use of pyrolysis dates back to ancient Egyptian times, when they were using pyrolysis for the preparation of tar for caulking boats and as an embalming agent [2]. In recent years, it has regained popularity in an effort to decrease the carbon footprint and identify alternative sources of energy and value-added chemicals from wood waste [3]. In the early 1980s, the pyrolysis process was improved and researchers were able to obtain better yields using a fast pyrolysis, in which a biomass feedstock is heated at a rapid rate and the vapors produced are also condensed rapidly [2,4].

Fast pyrolysis is regarded as one of the most promising thermo-chemical conversion techniques for producing liquid biofuels [4]. In addition, fast pyrolysis has a number of advantages over other competing technologies, such as high thermal efficiency, estimated low production cost, and low fossil fuel inputs [4].

Pyrolysis is able to provide material that has a wide range of qualities for a variety of applications [4,5]. Bio-oil derived from fast pyrolysis is a complex mixture of high energetic density and is composed of carbohydrates and phenols set free from lignin, hemicellulose, and cellulose during the process of thermal-degradation [6]. Bio-oil has a potential to be used for (i) energetic purpose; (ii) as a raw material for industry; and (iii) a source of value-added biochemical and bio-products [7].

Pyrolytic lignin derived from fast pyrolysis is a very complex phenolic polymer that can be obtained from agriculture and forestry residues. In general, lignins are classified as three-dimensional amorphous polymers consisting of methoxylated phenylpropane structures [8]. The important functional groups in lignin molecule include (i) hydroxyl; (ii) methoxyl; (iii) carbonyl; and (iv) carboxyl groups. The numbers and proportions of these groups can vary in the lignins depending upon the extraction process as well as the feedstock [9].

Despite the significance of lignin as a valuable bio-polymer, its real industrial potential has not been developed. In order to develop new practical applications from lignin, there is strong need to characterize its chemical reactivity and functional properties [10] that are important for its bioactivity and antioxidant potential. Several investigators have reported potential health benefits associated with lignins, such as its antitumor, antibacterial, antiparasitic, antiviral, and immunopotentiating and antioxidant activities [11,12,13,14,15,16,17,18].

Lignins have antioxidant potential, which is their capacity to quench the production of free radicals, and their ability to stabilize free radicals induced by oxygen and its radical species. A better understanding of natural antioxidants from pyrolytic lignins will facilitate their use in the food industry and as feedstock as value-added chemicals to preserve food, colour, flavor, and active vitamin contents [19]. The increased demand for natural food additives, sustainable use of resources and consumer demand for natural ingredients is prompting food manufacturers to replace synthetic antioxidants with natural antioxidant compounds that are regarded as safe with no health risk to the consumers [20]. Lignin is an underutilized component of renewable plant biomass, which is a rich resource of phenolics and holds a potential to be used as a natural source of antioxidants. This investigation was initiated with the aim to study the antioxidants from pyrolytic lignins. The 2,2-diphenyl-1-picrylhydrazyl scavenging activity (DPPH), trolox equivalent antioxidant capacity (TEAC) and total phenolics content (TPC) assays were identified as suitable methods for the measurement of antioxidant activity in a variety of pyrolytic lignins and identification of key components achieved using HPLC-MS/MS and untargeted metabolomics.

## 2. Results and Discussion

### 2.1. HPLC-M/MS and Metabolomic Analysis

**Figure 1:**
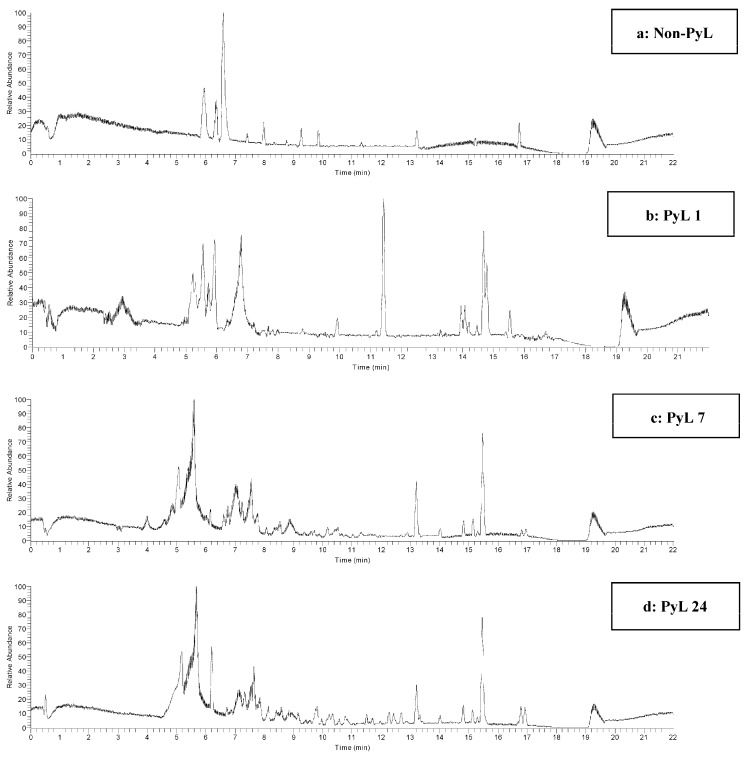
Selected HPLC-MS chromatograms (base peak) obtained using LTQ-ORBITRAP (Discovery) in the positive mode: (**a**) non-PyL; (**b**) PyL 1; (**c**) PyL 7; and (**d**) PyL 24, revealing the diversity of separated compounds from selected samples.

**Figure 2:**
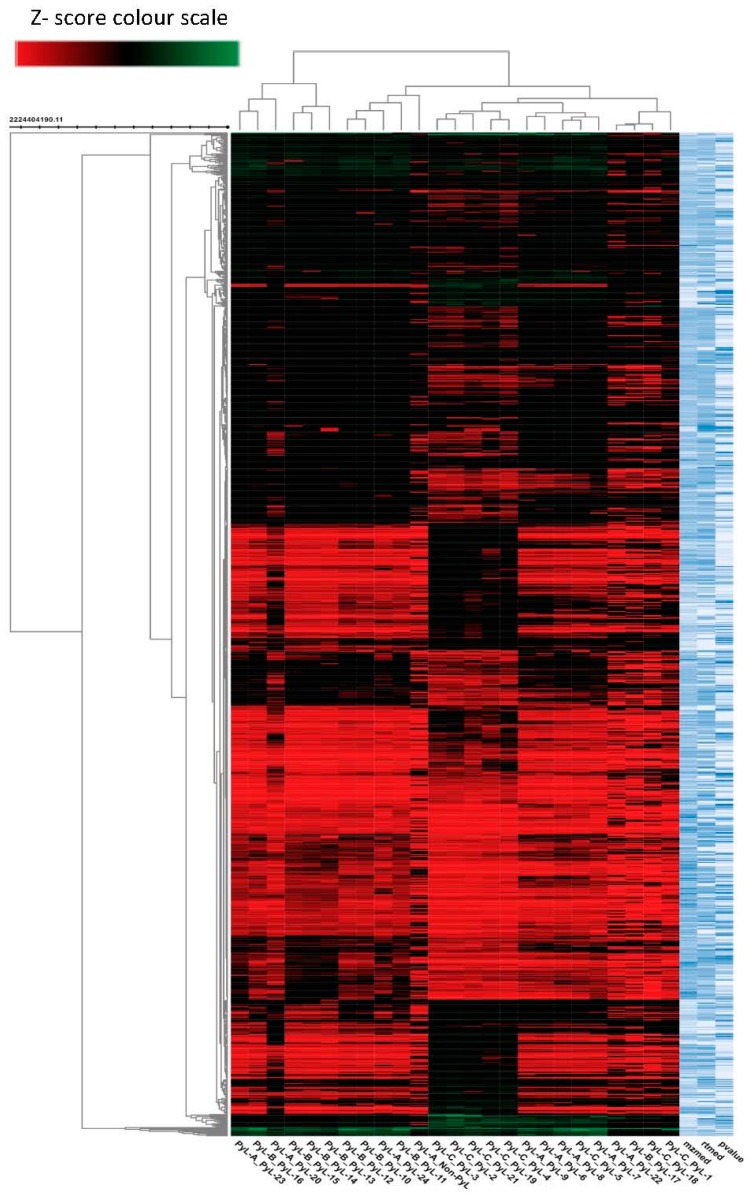
The heat map (dendrogram view) of the pyrolytic lignins, generated using the XCMS bioinformatics platform, showing the comparison of metabolite features between different samples. In dendrogram, each row represents a metabolite feature and each column represents samples. Metabolite features, the level of which varies significantly (*p* < 0.05) are projected on the heat map and were used for sample clustering. The row *Z* score (scaled expression) value of each feature is plotted in red (high abundance) to green colours (low abundance).

Thermal breakdown of lignins produces polyphenolic compounds that have a potential to be used as natural antioxidants. HPLC-MS/MS base peak chromatograms revealed (Figure 1) that selected samples have varying level of phenolic compounds. Phenolic profiles obtained in order of their decreasing complexity are samples PyL 24, PyL 7, PyL 1, and non-PyL. The putative identification of the compounds from pyrolytic lignins was determined using an untargeted metabolomics approach for metabolite identification (XCMS). In addition, spectral analysis and chromatography with standards was also used for compound identification (where applicable). High-throughput metabolomic analysis revealed the presence of several compounds with antioxidant and antimicrobial properties such as 2-(4-Allyl-2-methoxyphenoxy)-1-(4-hydroxy-3-methoxyphenyl)-1-propanol, benzyl benzoate, fisetinidol, phenyllactic acid, 2-phenylpropionic acid, and vanillin (Table 1). The data also indicated that the percent peak area of compounds obtained using XCalibur (Version 2.2, Thermo Scientific, San Jose, CA, USA) is higher in pyrolytic kraft lignins in contrast to non-pyrolytic kraft lignin. Currently little is known about the metabolite distribution of pyrolytic lignins derived from fast pyrolysis, as it depends upon multiple parameters (e.g., the feedstock and operating conditions of the reactor). Given the complexity of the data, we have used a non-targeted metabolomic approach using XCMS bioinformatics platform to obtain a global picture of the metabolites from pyrolytic lignins. The XCMS platform allowed for multigroup comparison, and it can be evident from heat map dendrogram (Figure 2) that the different samples of pyrolytic lignins can be clustered into five groups, implying the influence of metabolite features of the samples with high clustering coefficient. The untargeted profiling and metadata analysis using XCMS resulted in the detection of 1000 metabolite features (*p* < 0.05) from the pyrolytic lignins. The significance of the heat map lies in its unique ability to identify clusters of samples with similar metabolic patterns and discriminating metabolites that can drive the sample clustering [21]. The cloud map (Supplementary Materials) generated during this analysis allowed the detection of metabolite features (based on its retention time and *m*/*z*), whose levels differ between different groups (*p* < 0.01). The interactive feature of cloud map of XCMS platform allowed the detection of certain metabolites with high statistical confidence level (*p* < 0.001), including fisentinidol, carajurin, and gibberellin A9. Overall, the untargeted metabolic approach of XCMS bioinformatics platform provided an efficient means of high-throughput metabolic profiling and metadata analyses of complex samples for comparative analysis.

### 2.2. Antioxidants and Total Phenolics Analysis

The 2,2-diphenyl-1-picrylhydrazyl assay (DPPH), 2,2′-azino-bis (3-ethylbenzothiazoline-6-sulphonic acid (ABTS) or trolox equivalent antioxidant capacity assay (TEAC), and total phenolics content (TPC) were employed in this study because of their wide utility in the determination of antioxidants from food and feed sources [22].

**Figure 3:**
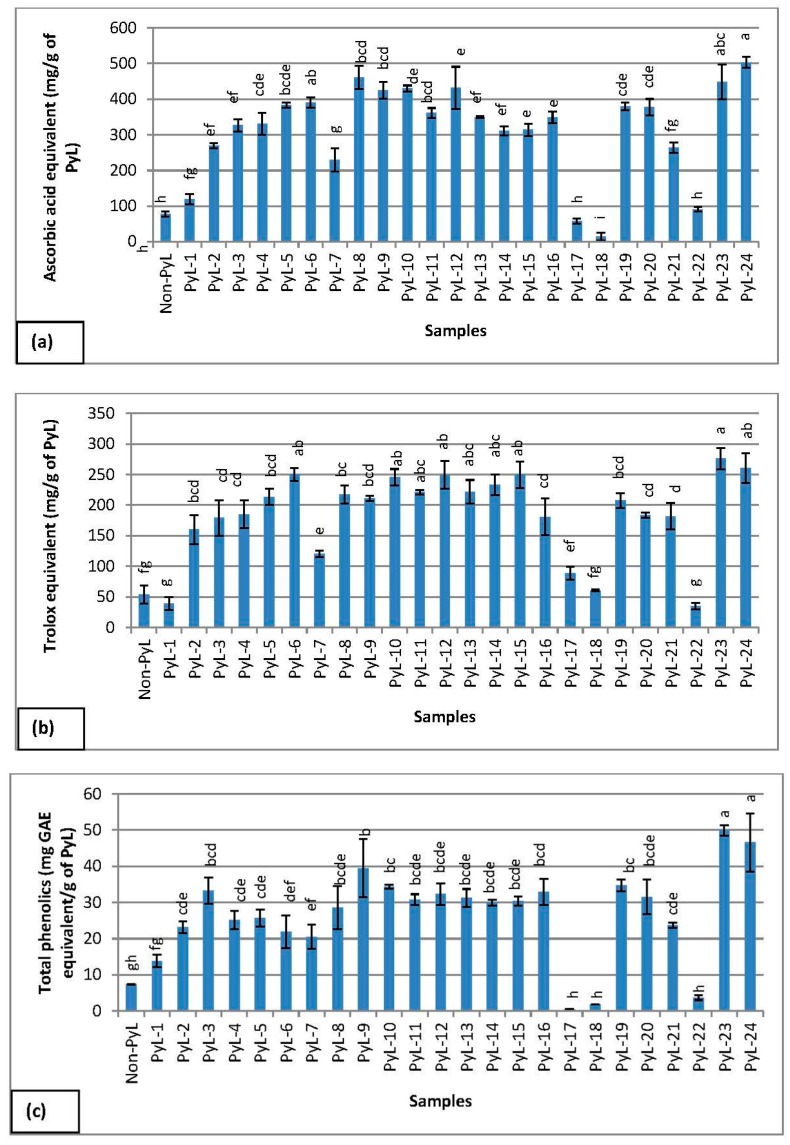
Antioxidant activity of pyrolytic lignins, as determined by (**a**) DPPH assay; (**b**) TEAC assay; and (**c**) total phenolics assay. Results are expressed: (**a**) mg ascorbic acid equivalents/g of pyrolytic lignin (PyL); (**b**) mg trolox equivalents/g of pyrolytic lignin (PyL); and (**c**) mg gallic acid equivalents (GAE)/g of pyrolytic lignin (PyL). Each value is presented as a mean ± SD (*n* = 3). Analysis of variance was performed using one-way completely-randomized ANOVA (CoStat version 6.4) and means are separated using the Student-Newman-Keuls test (*p* < 0.05). The bars with same alphabet superscript are not significantly different.

Pyrolytic lignins were evaluated for radical scavenging activity using DPPH assay, the results (Figure 3a) show that the samples have wide range of radical scavenging activity between 14 and 503 mg ascorbic acid equivalents/g of pyrolytic lignin. The level of significance between different samples was determined using one-way completely-randomized ANOVA, where means were separated using Student-Newman-Keuls test (*p* < 0.05). The results also showed that the high antioxidant activity was evident in pyrolytic lignins in contrast to non-pyrolytic lignin (non-PyL). These data can be complemented by HPLC-MS/MS analysis that revealed the differences in the polyphenolic compounds. A possible explanation for the higher antioxidant activity of pyrolytic lignin is due to its phenolic hydroxyl groups [23] and lower methoxyls (MeO) groups in comparison to non-pyrolytic lignin [24]. In this context, Nsimba and co-workers proposed that thermolytic cleavage of the methoxyl group of the guaiacyl ring can decrease the methoxyl content in pyrolytic lignins, the lack of methoxyls in the pyrolytic product suggest its chemical structure closer to H-(*p*-hydroxyphenyl) type lignin that results in the increase of its antioxidant capacity [25]. The difference in the radical scavenging activity in pyrolytic lignins also reflects the difference in the phenolic groups that may form during the different treatments/processes.

The trolox antioxidant capacity method is based on the ability of an antioxidant molecules to quench ABTS^•+^ radical. The ABTS^•+^ radical scavenging activity was quantified for a variety of pyrolytic lignins and a significant variation in the antioxidant activity (35–277 mg trolox equivalents/g of pyrolytic lignin) was evident from the different samples (Figure 3b). The level of significance between different samples was determined using one-way completely-randomized ANOVA. The high ABTS^•+^ free radical scavenging activity was observed in pyrolytic lignins in contrast to non-pyrolytic lignin. The varying level of antioxidant activity observed in pyrolytic lignins is consistent with HPLC-MS/MS data, which showed less phenolic compounds (Figure 1 and Table 1). The low antioxidant activity was observed in other samples (PyL 17, 18, and 22) that are also in low compound amounts determined by HPLC-MS/MS (data not shown). The radical scavenging activity of phenolic groups in lignin compounds observed with the ABTS^•+^ radical is mainly due to electron or proton transfer mechanisms. Conversely, the antioxidant effect of lignin on DPPH radical is due to the combination of electron transfer, as well as H atom [26]. The higher antioxidant activity of pyrolytic lignin can be explained by its high aromaticitiy [24] and a lower molecular weight in contrast to non-pyrolytic lignin [27,28].

We have also determined the total phenolics content in the pyrolytic lignins. Quantification of phenols in pyrolytic lignins is very important because phenols influence the reactivity and stability of the pyrolytic lignins. Furthermore, it is one of the important products derived from the pyrolytic lignins and is considered as a phenolic replacement in phenol-formaldehyde resins [29]. The total phenolics content was variable between different pyrolytic lignins, between 0.42 and 50 mg GAE/g of pyrolytic lignin (Figure 3c), where the level of significance was determined using one-way completely-randomized ANOVA (*p* < 0.05). The high total phenolics content were observed in pyrolytic lignins, in contrast to non-pyrolytic lignins. The low phenolics content was also evident in some samples (PyL 17, 18, and 22), these results are consistent with HPLC-MS/MS analysis (data not presented) that show less phenolics compound. The free phenolic hydroxyl groups in lignin compounds are crucial for its antioxidant activity, however, the aliphatic hydroxyl groups have an opposite effect on its radical scavenging activity [30]. Similarly, Nsimba and coworkers [25] have reported the increase of phenolic hydroxyls (ArOH) and aliphatic hydroxyls (AlkOH) group of pyrolytic lignins, whereas they also reported the loss of MeO on the pyrolytic lignins. It is plausible that pyrolysis would have promoted the cleavage of the α- and β-ether linkages between lignin subunits and resulted in the formation of new ArOH and AlkOH groups, as reported for pyrolytic EteK lignins [25]. Conversely, during the pyrolysis, the thermolytic cleavage of the methoxyl group of the guaiacyl ring can also result in the decrease of methoxyl groups of pyrolytic lignin [25].

### 2.3. Principal Component Analysis (PCA)

**Figure 4:**
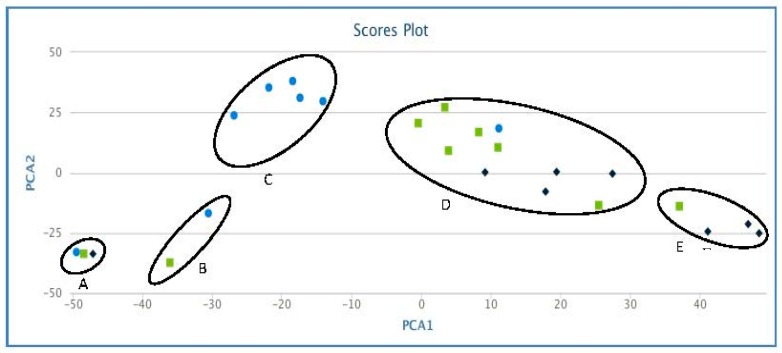
Principal component analysis (PCA), revealing the correlations between a variety of pyrolytic lignins (derived from fast pyrolysis). PCA plot was generated using XCMS bioinformatics platform for metabolomics. Clusters: Group A phenolics content (1–5 mg gallic acid equivalents (GAE)/g of pyrolytic lignin (PyL), Group B phenolics content (5–15 mg GAE equivalents/g of PyL), Group C, D, and E phenolics content (≥15 mg GAE equivalents/g of PyL).

To show the relationship between samples, we used principal component analysis (PCA), which is one of the most widely used multivariate analysis tools in metabolomics, especially in untargeted metabolic profiling or fingerprinting [31]. The PCA revealed the relationship between pyrolytic lignins that makes use of covariance or correlations among variable or metabolite features. Five main clusters were observed on the PCA plot, implying the influence of metabolic features. It can be appreciated (Figure 4) that clustering of samples on PCA plot has some degree of influence of phenolics content in clustering of the pyrolytic lignins. The samples with lowest (1–5 mg) phenolics content (PyL 17, 18, and 22) clustered in group A, whereas, samples with intermediate (5–15 mg) phenolics content (non-PyL and PyL1) form group B. On the other hand, pyrolytic lignins with higher phenolics content (≥15 mg) form three clusters, C, D, and E. The results imply the significance of phenolics content in the clustering coefficient of samples and determining the properties of pyrolytic lignins.

### 2.4. Correlation Analysis

**Figure 5:**
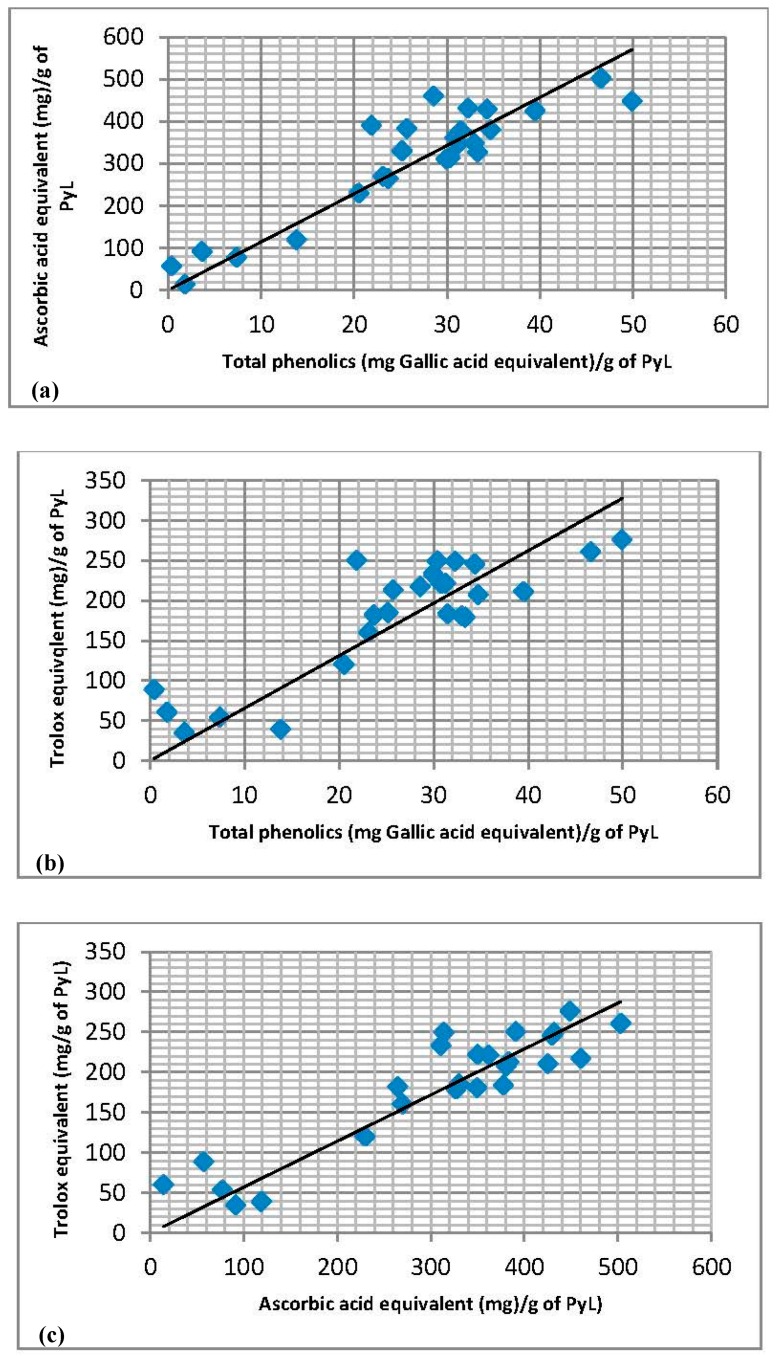
Correlation between antioxidant assays and total phenolics content of pyrolytic lignins (PyL), (**a**) DPPH versus TPC, (*r* = 0.8663, *p* < 0.0001); (**b**) TEAC versus TPC (*r* = 0.8044, *p* < 0.0001); and (**c**) DPPH versus TEAC (*r* = 0.8851, *p* < 0.0001). Product moment correlation was performed using CoStat (version 6.4).

In order to understand the relationship between pyrolytic lignins, their radical scavenging effect, total antioxidant capacity and total phenolics content, a correlation analysis was performed using Pearson product moment correlation. A very significant correlation (Figure 5a–c) was observed between the total phenolics (TPC) and TEAC (*p* ≤ 0.0001), TPC and DPPH (*p* ≤ 0.0001) and DPPH and TEAC (*p* ≤ 0.0001). The high values of the correlation coefficient were obtained for DPPH versus TEAC (*r* = 0.8851), DPPH versus TPC (*r* = 0.8663), and TEAC versus TPC (*r* = 0.8044). These results imply that the phenolics content in the pyrolytic lignins are very important in determining its radical scavenging activity and antioxidant properties. Furthermore, total phenolics content of a pyrolytic lignins can serve as a useful indicator for the antioxidant activity of pyrolyic lignins derived from fast pyrolysis.

## 3. Materials and Methods

### 3.1. Reagents

Ascorbic acid, potassium persulfate, gallic acid, DPPH (2,2-diphenyl-1-picryhydrazyl), 2,2-azino-bis(3-ethylbenzothiazoline)-6 sulphonic acid (ABTS) and Folin-Ciocalteu’s phenol reagent were purchased from Sigma-Aldrich (St. Louis, MO, USA). Methanol (LC/MS grade) and ultra-pure water (Fisher Scientific, Ottawa, ON, Canada) was used throughout the experiments. All other reagents were of analytical grade.

### 3.2. Pyrolytic Kraft Lignins

The Kraft lignin was extracted from black liquor in Weyerhaeuser Canada′s Grande Prairie Mill (Grande Prairie, AB, Canada). The feedstock contained 3.6% of moisture, 55.8% of volatile matter, 40.3% of fixed carbon, and 0.3% of ash. The elemental composition was 62.4% of carbon, 6.1% of hydrogen, 0.2% of nitrogen, 29.1% of oxygen, and 2.0% of sulfur, on a dry biomass basis. The higher heating value of the lignin powder was 25.8 MJ/kg [32]. Pyrolytic kraft lignins used in this study were obtained from Institute for Chemical and Fuels from Alternative Resources, University of Western Ontario. Pyrolytic lignins were collected from a bubbling fluidized bed pyrolysis reactor equipped with a fractional condensation train [32]. Pyrolytic lignins were dissolved in methanol (20 mg/mL) and were filtered through 0.22 µm GHP filters (Acrodisc Syringe Filters, Pall Corporation, Saint-Laurent, QC, Canada). Samples were used with appropriated dilution for antioxidant analysis and total phenolics content, as explained in Section 3.4.

### 3.3. HPLC-MS/MS (ORBITRAP)

High-performance liquid chromatography was performed using a Thermo Scientific LTQ Orbitrap Discovery (MS 2.5.5, Thermo Scientific, San Jose, CA, USA) equipped with an Autosampler Accela AS 2.2.1, and pump 1.04.05. The instrument was equipped with a Syncronis C18, Thermo Scientific column: 50 mm length, 2.1 mm I.D., and 1.7 µm particle size that was operating at room temperature. The injection volume was 10 µL. A gradient technique was employed in this study with a flow rate of 0.2 mL/min. Solvent A was composed of AcN acidified with 0.1 vol % of formic acid, whereas solvent B was composed of water acidified with 0.1 vol % of formic acid. The gradient was programmed as follows: solvent A 2 vol %, increased to 10 vol % at 2 min, increased to 25 vol % at 6 min, increased to 50 vol % at 10 min, increased to 75 vol % at 14 min, increased to 95 vol % at 18 min, decreased to 2 vol % at 20 min, followed by 2 min of isocratic elution with 2% of solvent A (total elution time 22 min). The LTQ Orbitrap MS was equipped with an ESI source operating in positive ionization mode using the following operating parameters: electrospray voltage of 3.1 kV, sheath gas flow rate of 8 abu (arbitrary unity), auxiliary gas flow rate of 1 abu, capillary temperature of 270 °C, capillary voltage set to 49.00 V, and tube lens offset at −148.43 V. Instrument calibration was performed externally prior to running each sequence, employing the “Thermo Scientific Pierce LTQ Velos ESI positive ion calibration solutions”. Accurate mass spectra of [M + H]^+^ ions were recorded from 100 to 1000 *m*/*z*, the mass resolution power of the mass analyzer was set to 30,000 (m/∆m) at *m*/*z* 400. Nitrogen gas (purity 99.95%) was used both as sheath gas and auxiliary gas, serving as the co-collision gas in the HCD cell and the bath gas in the C-trap.

#### Data Analysis

Data were analyzed using multi-group method of XCMS online (https://xcmsonline.scripps.edu) bioinformatics platform [33]. The HPLC-MS/MS raw data files from ORBITRAP (Discovery, Thermo Scientific, San Jose, CA, USA) were converted to mzXML format using Proteowizard, and were subsequently processed for peak detection, retention time correction, chromatogram alignment, metabolite feature metadata/statistical analysis, and putative identification using METLIN database. The parameter settings for XCMS processing were as follows: centWave for feature detection (∆*m*/*z* = 15 ppm, minimum peak width = 10 s and maximum peak width = 120 s); obiwarp settings for retention-time correction (prof Step = 1); and parameters for chromatogram alignment, including mzwid = 0.015, minfrac = 0.5 and bw = 5. The relative quantification of metabolite features was based on peak areas [34].

### 3.4. Antioxidant Activity Determination

Antioxidant activities of the pyrolytic lignins were determined using three methods (i) 2,2-diphenyl-1-picrylhydrazyl scavenging effect (DPPH); (ii) trolox equivalent antioxidant capacity assay (TEAC); and (iii) total phenolics assay (TPC). TEAC and TPC are an electron transfer (ET) based assays, whereas DPPH is mainly ET based and has a hydrogen-atom abstraction mechanism (HAT) [35]. Due to their different mode of actions (ET or HAT), these methods have been successfully employed to study antioxidant activities of plant extracts in the complex media [35,36,37]. During this study, modified microplate assays were employed in order to minimize the use of sample, chemicals, and waste disposal.

#### 3.4.1. DPPH (2,2-diphenyl-1-picrylhydrazyl) Scavenging Effect

DPPH radical-scavenging activity was determined using by microplate assay with some modifications of Yu and coworkers [38]. Briefly, 150 µL of DPPH dissolved in methanol (100 µM) was added to 150 µL of standard or sample (different concentrations), prepared in methanol. The mixture was then shaken, and subsequently incubated in the dark for 30 min at room temperature. Finally, the decrease in absorbance of DPPH was measured at 517 nm using a TECAN plate reader at room temperature. The remaining concentration of DPPH was determined using a calibration curve of ascorbic acid. The results were expressed as mg ascorbic acid equivalents/g of pyrolytic lignin.

#### 3.4.2. TEAC (Trolox Equivalent Antioxidant Capacity) Assay

The ABTS^•+^ radical scavenging activity of pyrolytic lignins were determined after Re and coworkers [39] with some modifications. The TEAC method is based on the capacity of the antioxidants to quench ABTS^•+^, which is a blue-green chromophore and has a characteristic absorption at 734 nm. ABTS^•+^ solution was prepared, thereby mixing ABTS salt (7 mM) with potassium persulfate (2.45 mM). The solution was prepared 12–16 h before use and was stored in the dark at room temperature. The ABTS^•+^ solution was diluted with methanol to obtain an absorbance of 0.70 (±0.002) at 734 nm. In general, the addition of antioxidants to the preformed radical cation decolourizes the ABTS^•+^, which is then reduced to ABTS. Standards and samples (of appropriate dilution) were prepared in methanol at various concentrations and 300 µL of ABTS^•+^ was added to the reaction mixture. The reaction contents were incubated in TECAN plate reader at 25 °C and readings were obtained after 6 min. The results were expressed as mg trolox equivalents/g of pyrolytic lignin. Trolox (6-hydroxy-2,5,7,8-tetramethylchroman-2-carboxylic acid) was used as a standard.

#### 3.4.3. Total Phenolics Content (TPC) Analysis

Total phenolics content (TPC) of the pyrolytic lignins was determined after Singleton and coworkers [40] with some modifications. Briefly, 2 µL of sample (with appropriate dilution) or standard was added to microplate well, and then 158 µL of MilliQ water was added to each sample. Next, 10 µL of Folin-Ciocalteu phenol reagent was added and microplate was mixed by swirling actions. After 8 min of interval, 30 µL of sodium carbonate was added, and subsequently plate was incubated at room temperature in dark for 2 h. Finally, microplate was read at 765 nm and TPC was calculated using Equation (1) and expressed as mg GAE/g of pyrolytic lignin. Gallic acid was used as a standard.

T = C × V/M(1)

where T is the total phenolics content in mg/g of the extracts as GAE. C is the concentration of gallic acid established from the calibration curve in mg/mL, V is the volume of the extract solution in mL, and M is the weight of the extract in g.

### 3.5. Statistical Analysis

All assays were performed in triplicates and the results obtained were analyzed using one-way analysis of variance (ANOVA) to compare the data (mean). Furthermore, means were separated using the Student-Newan-Keul’s test, where *p* < 0.05. In addition, correlation analysis between DPPH, TEAC and TPC was performed using the Pearson product moment correlation method. CoStat (Version 6.4, CoHort Software, Monterey, CA, USA) was used for the statistical analysis.

## 4. Conclusions

The current study provides a first report on the HPLC-MS/MS combined with untargeted metabolomic analysis of pyrolytic lignins. The untargeted metabolomics provided a useful tool for the rapid identification of metabolites from pyrolytic lignins. The HPLC-MS/MS and untargeted metabolomics revealed that pyrolytic lignins have high compound amounts and high antioxidant activity (as determined by antioxidant assays) in contrast to non-pyrolytic lignins. Over all, the autonomous untargeted metabolomics provided an efficient means of high-throughput metabolomic profiling of lignins derived from fluidized-bed fast pyrolysis. Pyrolytic lignins contain natural antioxidants that can be used as a source of value-added chemicals. The results also show that the pyrolytic lignins have a varying level of antioxidant activity, which is related to the presence of phenolic compounds. It is likely that novel compound from pyrolytic lignins may be extracted and can be used as natural antioxidants in the food, pharmaceutical and nutraceutical industry. Lignin-derived value-added chemicals can provide food additives that are natural, safe, and acceptable by consumers. Furthermore, TEAC, DPPH, and TPC assays are suitable methods for the analysis of antioxidant activity of pyrolytic lignins.

## Figures and Tables

**Table 1:** HPLC-MS/MS and metabolomic analysis (XCMS Bioinformatics Platform) was used for the putative identification of compounds from pyrolytic lignins. Formula, actual mass, M + H, Ring and Double-Bond (RDB), Retention Time (RT), and peak area (%) among reported compounds.

Identified Compound	Actual Mass	M + H	Formula	RDB	RT	Peak Area (%)			
						Non-PyL	PyL 1	PyL 7	PyL 24
2-(4-Allyl-2-methoxyphenoxy)-1-(4-hydroxy-3-methoxyphenyl)-1-propanol	344.1623739	345.1693	C_20_H_25_O_5_	8.5	10.78	N.I.	0.16	0.34	1.01
Coumarinic acid	178.0629942	179.0701	C_10_H_11_O_3_	5.5	3.76	N.I.	N.I.	0.48	0.13
Phenyllactic acid	166.062991	167.0703	C_9_H_11_O_3_	4.5	6.16	N.I.	N.I.	0.79	4.45
(+/−)-3-[(2-methyl-3-furyl)thio]-2-butanone	184.0558003	185.063	CH_9_O_5_N_6_	0.5	13.86	N.I.	N.I.	0.5	0.06
2,5-Dibutyl-4-methyloxazole	195.1623143	196.1695	C_12_H_22_ON	2.5	15.23	0.52	0.53	0.38	0.38
2-Phenylpropionic acid	150.0681	151.0754	C_9_H_11_O_2_	4.5	7.24	N.I.	0.13	1.25	3.72
6,3′-Dimethoxyflavone	282.29	283.1	C_17_H_15_O_4_	10.5	8.9	N.I.	0.3	N.I	1.22
Thymol	150.1044651	151.1117	C_10_H_15_O	3.5	10.47	N.I.	0.2	2.11	0.39
2,4-hexadienal	96.05751488	97.0648	C_6_H_9_O	2.5	2.64	N.I.	6	1.05	0.02
Dehydrocostus lactone	230.1306798	231.1379	C_15_H_19_O_2_	6.5	10.36	N.I.	N.I.	N.I	0.83
7-Hydroxyflavan	226.0993797	227.1066	C_15_H_15_O_2_	8.5	12.88	N.I.	N.I.	0.42	0.25
Benzyl benzoate	212.0837296	213.0909	C_14_H_13_O_2_	8.5	11.53	N.I.	N.I.	0.5	0.57
Vanillin *	152.15	153.05	C_8_H_9_O_3_	4.5	5.16	N.I.	5.61	0.19	8.07
Fisetinidol	274.0841235	275.091	C_15_H_15_O_5_	8.5	7.7	N.I.	N.I.	1.7	5.44

Note: (a) * Compound identified using authentic standard; (b) Not identified, N.I.

## Supplementary Materials

Supplementary materials are available online. Figure S1: Test cloud plot of metabolite features, the level of which varies significantly (*p* < 0.01) between pyrolytic lignins derived from fast pyrolysis. The metabolite features are represented by bubbles on the cloud plot depending upon their retention time (*x*-axis) and *m*/*z* (*y*-axis). Each bubble represents a metabolite feature, where the size of the bubble denotes its intensity, and the colour of each bubble (from light blue to dark grey), indicates its statistical significance (*p* < 0.01).
