# Supplementary material for: Antioxidant Activity of the Lignins Derived from Fluidized-Bed Fast Pyrolysis
Source: Molecules. 2017 Mar 1;22(3):372. doi: 10.3390/molecules22030372 (PMC6155384; doi:10.3390/molecules22030372)
Supplement: Supplementary file 1 [file molecules-22-00372-s001.pdf]

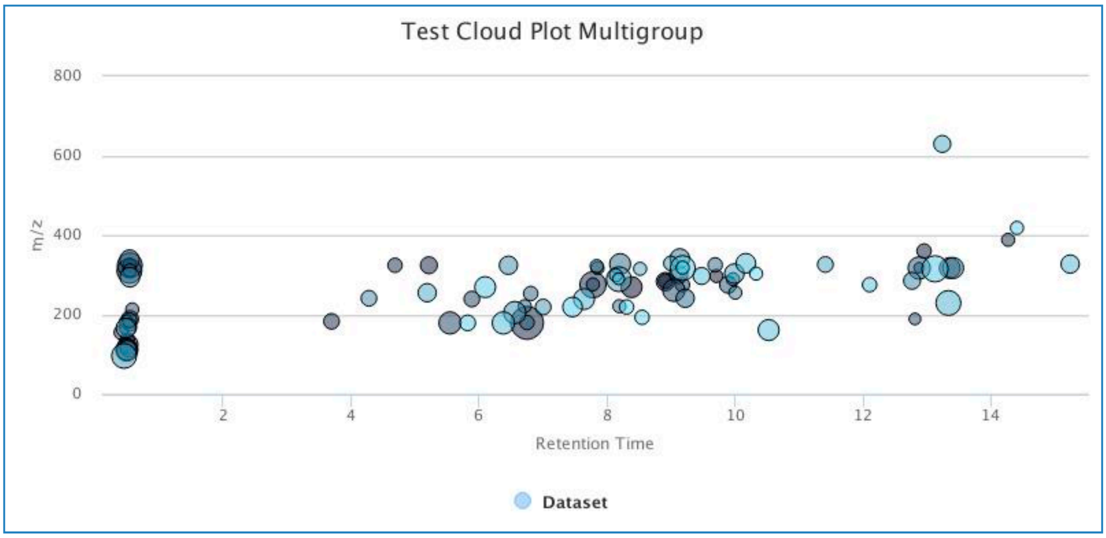

Figure S1. Test cloud plot of metabolite features, the level of which varies significantly ($p < 0.01$) between pyrolytic lignins derived from fast pyrolysis. The metabolite features are represented by bubbles on the cloud plot depending upon their retention time (x-axis) and m/z (y-axis). Each bubble represents a metabolite feature, where the size of the bubble denotes its intensity, and the colour of each bubble (from light blue to dark grey), indicates its statistical significance ($p < 0.01$).
